# The correlation between serum 25-hydroxy-vitamin D levels and anti-SARS-CoV-2 S-RBD IgG and neutralizing antibody levels among cancer patients receiving COVID-19 vaccines

**DOI:** 10.3389/fnut.2022.1066411

**Published:** 2022-12-13

**Authors:** Andhika Rachman, Anggraini Iriani, Dimas Priantono, Bayu Bijaksana Rumondor, Rachelle Betsy, Samuel Juanputra

**Affiliations:** ^1^Division of Hematology and Medical Oncology, Department of Internal Medicine, Dr. Cipto Mangunkusumo General Hospital – Faculty of Medicine Universitas Indonesia, Jakarta, Indonesia; ^2^Department of Clinical Pathology, YARSI University, Jakarta, Indonesia; ^3^Department of Internal Medicine, Dr. Cipto Mangunkusumo General Hospital - Faculty of Medicine Universitas Indonesia, Jakarta, Indonesia

**Keywords:** vitamin D, cancer, vaccine, SARS-CoV-2, COVID-19, S-RBD IgG, neutralizing antibody

## Abstract

**Introduction:**

During the coronavirus disease 2019 (COVID-19) pandemic, vitamin D has been established as an immune-modulator that reduces pro-inflammatory damage which effectively diminish the severity of COVID-19. Vitamin D also has a significant effect against influenza and dengue and increase the seroconversion following influenza vaccination. To date, the role of vitamin D in optimizing the efficacy of COVID-19 vaccines remains unclear. This study aimed to analyze the correlation between serum 25-hydroxy-cholecalciferol or 25(OH)D levels and anti-SARS-CoV-2 S-RBD IgG and neutralizing antibody levels among cancer patients.

**Methodology:**

A multicenter cross-sectional study was conducted among solid and hematologic cancer patients who were vaccinated with two doses of the same types of COVID-19 vaccines (either mRNA, non-replicating viral vector, or inactivated) within 6 months.

**Result:**

The median serum 25(OH)D level in 119 cancer patients was 36.36 [IQR = 30.30] ng/mL. The seropositivity of S-RBD IgG and NAb reached 93.3 and 94.1%, respectively. The S-RBD IgG level was significantly higher in the sufficient group (median = 414.07 [1,441.83] AU/mL) than in the deficient group (median = 91.56 [652.00] AU/mL) (*p*-value = 0.049). Among non-chemotherapy subjects, the anti-SARS-CoV-2 S-RBD IgG levels had a significant positive correlation with 25(OH)D levels (*p*-value = 0.03; *R* = 0.588). The NAb levels also showed significantly positive correlation with 25(OH)D level (*p*-value = 0.005; *R* = 0.561). The 25(OH)D levels were positively correlated with S-RBD IgG levels among subjects younger than 60 years old (*p*-value = 0.047; *R* = 0.136). However, serum 25 (OH)D levels showed no such correlation with S-RBD IgG levels among subjects older than 60 years old (*p*-value = 0.933; *R* = 0.136).

**Conclusion:**

Both anti-SARS-CoV-2 S-RBD IgG and NAb levels developed moderate correlation with 25(OH)D levels among subjects treated without chemotherapy. The S-RBD IgG levels also had positive correlation with 25(OH)D levels among subjects younger than 60 years old. Thus, we recommended cancer patients to maintain serum 25(OH)D levels above 30 ng/mL (75 nmol/L) to enhance the efficacy of COVID-19 vaccines.

## Introduction

Coronavirus disease 2019 (COVID-19) is a respiratory and systemic illness caused by a novel coronavirus (nCoV) and is identified as severe acute respiratory syndrome coronavirus 2 (SARS-CoV-2) (1). During the COVID-19 pandemic, vitamin D acts as an immune modulator and has antioxidant properties that reduce the damage caused by pro-inflammatory cytokines through a variety of mechanisms, including modulating the ACE-2 receptors, maintaining pulmonary barrier function, and increasing neutrophil activity (2–4). At the early viremia stage, this vitamin improves the innate immune response and turns the adaptive immune response toward T helper cell-2 (Th2) type (2, 5). Hence, it has been established that daily vitamin D_3_ supplementation effectively prevents acute respiratory infections and diminishes the severity of COVID-19 (2, 6, 7, 8, 9). Prior research has found that vitamin D_3_ (cholecalciferol) supplementation has a significant effect against various viral diseases, including influenza and dengue (10, 11). A seroconversion following an influenza vaccination was higher among people with higher 25(OH)D levels (12). However, the role of vitamin D in optimizing the efficacy of COVID-19 vaccines remains unclear (10, 12).

Vaccine is the most powerful strategy to limit the extent of SARS-CoV-2 infection. Vaccine designates a potent strategy for reducing COVID-19 morbidity (13, 14). Nanoparticle-based SARS-CoV-2 vaccines, such as inactivated, non-replicating viral vector, and mRNA vaccines, have already been applied clinically worldwide (12, 15, 16). These COVID-19 vaccines can induce both SARS-CoV-2 specific CD4+ and CD8+ T-cell responses and neutralizing antibody production (15, 17–19).

Cancer patients have a higher risk of severe COVID-19 outcomes, which may lead to a higher morbidity and mortality rate. Thus, despite being initially excluded from the pivotal clinical trial of the COVID-19 vaccine, they were considered a high-priority group to vaccinate (20–24). A systematic review revealed that cancer patients had a lower seroconversion rate compared to healthy subjects (25). Thus, the use of adjuvant strategies with vitamin D_3_ supplementation to improve responses to COVID-19 vaccines seems to be advantageous in cancer patients (10, 26).

We hypothesized that the use of vitamin D_3_ supplements can potentially improve the immune responses from COVID-19 vaccines. Prior studies have established that vitamin D has significant biological effects on both the innate and adaptive immunity, which are expressed by a multitude of immune cells such as lymphocytes, monocytes, macrophages, and dendritic cells (27–32). On the other hand, the COVID-19 vaccine induces the THαβ immune response and stimulates the development of long-term memory B cells, CD4 T cells, and CD8 T cells. Recent studies revealed that serum 25(OH)D levels are associated with these immunogenicity process (12).

Therefore, we expect that vitamin D has a beneficial role in enhancing the efficacy of COVID-19 vaccines as measured by the levels of anti-SARS-CoV-2 spike’s protein receptor binding domain immunoglobulin G (S-RBD IgG) and neutralizing antibody (NAb). The S-RBD IgG and NAb have been extensively used by researchers in phase I and phase II clinical trials to evaluate the efficacy and determine the optimal dose of COVID-19 vaccines (15, 33). Vitamin D_3_ is widely available and can be easily bought at over-the-counter pharmacies. We aim to analyze the correlation between 25(OH)D levels and anti-SARS-CoV-2 S-RBD IgG and neutralizing antibody levels among cancer patients after two-doses of COVID-19 vaccines, which is previously uncharted territory.

## Materials and methods

### Study design

This study was a multicenter cross-sectional study conducted at the Dr. Cipto Mangunkusumo General Hospital, Jakarta, Indonesia, and the Pondok Kopi Islamic Hospital, Jakarta, Indonesia. The samples in this study were gathered for 6 months, from October 2021 to March 2022. The included subjects were aged ≥ 18 years old; were diagnosed with either solid or hematologic cancers; and had received two-doses of COVID-19 vaccination without booster within 6 months before the evaluation. The COVID-19 vaccines were mRNA vaccines (BNT162b2, mRNA-1273), non-replicating viral vector vaccines (AZD1222), and inactivated vaccines (Coronavac, BBIBP-CorV vaccinations). Patients who already had their COVID-19 vaccine boosters were excluded.

### Measurement

The measurement of anti-SARS-CoV-2 S-RBD IgG and NAb serum levels was examined by Chemiluminescent immunoassay (CLIA) method using the Mindray immunoassay analyzer CL-900i. The results of both S-RBD IgG and NAb were measured in AU/mL. According to the assay manufacturer, the cut-off values for both S-RBD IgG and NAb seropositivity were greater than 10 AU/mL.

Serum 25-hydroxyvitamin D 25(OH)D levels were used to determine vitamin D status. Measurements were conducted by a competitive electrochemiluminescent protein binding assay using Cobas e411 from Roche Diagnostics. Following the second dose of the COVID-19 vaccine, serum 25(OH)D levels were measured.

### Statistical analysis

Extracted data was analyzed with Statistical Package for the Social Sciences (SPSS) version 27 for Macintosh. Any graph or plot were created using GraphPad Prism 9 for Macintosh. Bivariate analysis was conducted with either Pearson test or Kendall test for normally distributed data and not normally distributed data, respectively. The serum 25(OH)D levels between two subgroups were analyzed with either *t*-test or Mann-Whitney *U* test.

### Ethical approval

Ethical approval for this study was granted by the Ethics Committee of the Faculty of Medicine, Universitas Indonesia (ethical approval number: KET999/UN2.F1/ETIK/PPM.00.02/2021). This research was performed in accordance with the Declaration of Helsinki.

## Results

This cross-sectional study included 119 cancer subjects undergoing BNT162b2 (26.9%), mRNA-1273 (12.6%), AZD1222 (16%), and Coronavac or BBIBP-CorV (44.5%) vaccinations. The subjects gave written informed consent prior to recruitment. From the 119 subjects, 86.6% were female.

Approximately 92.4% of the participants were diagnosed with solid organ cancer, which included cancers of the brain, breast, gastrointestinal tract, head and neck, kidney, lung, pancreas, prostate, and testicles cancers. Hematologic malignancies, which include leukemia and lymphoma, were found in 7.6% of the participants (Table 1).

**TABLE 1:** Subject characteristics.

Characteristics	*N* (119)
**Age [*N* (%)]**	
≤60 years old	99 (83.2)
>60 years old	20 (16.8)
**Sex [*N* (%)]**	
Female	103 (86.6)
Male	16 (13.4)
**Cancer types [*N* (%)]**	
Hematologic	9 (7.6)
Solid	110 (92.4)
Serum 25(OH)D level, median [IQR] (ng/mL)	36.36 [30.30]
**Daily vitamin D_3_ supplementation [*N* (%)]**	
Yes	84 (70,59)
No	35 (29,41)
**Anti-SARS-CoV-2 antibody level (AU/mL)**	
S-RBD IgG, median [IQR]	270.56 [658.01]
NAb, median [IQR]	129.03 [225.61]
**Seropositivity [*N* (%)]**	
S-RBD IgG	111 (93.3%)
NAb	112 (94.1%)

IQR, interquartile range; SD, standard deviation; COVID-19, coronavirus disease 2019; S-RBD IgG, antibodies against receptor binding domain of SARS-CoV-2 spike protein; NAb, neutralizing antibody.

The chemotherapy regimens included anthracycline based chemotherapy, alkylating agents, antimetabolite drugs, hormonal therapies, kinase inhibitors and topoisomerase inhibitors, monoclonal antibodies, steroids, and vinca alkaloids. These regimens were used either as a single regimen or as a combined regimen according to National Comprehensive Cancer Network in oncology (NCCN) guidelines (34). According to the Charlson Comorbidity Index, subjects are considered to have a comorbidity when they have a history of at least one of the following conditions: diabetes mellitus, chronic kidney disease, cerebrovascular disease, dementia, peripheral vascular disease, chronic obstructive pulmonary disease (COPD), liver disease, peptic ulcer disease (PUD), connective tissue disease, myocardial infarction (MI), or congestive heart failure (CHF) (35). The included subjects had reached high seropositivity of anti-SARS-CoV-2 S-RBD IgG and NAb levels according to the assay manufacturer (Table 1).

A serum 25(OH)D level of less than 30 ng/mL is considered deficient by the Endocrine Society, the American Geriatric Society, and the National and International Osteoporosis Foundation (36). In this study, we divided serum 25(OH)D level into two categories, subjects with serum 25(OH)D levels < 30 ng/mL were considered deficient, whereas subjects with serum 25(OH)D levels ≥ 30 ng/mL were considered sufficient (Table 2).

**TABLE 2:** Vitamin D levels based on the subjects characteristics.

Subgroups	*N*	Serum 25(OH)D level (ng/mL)	
		Categories	Averages, in median [IQR] or mean ± SD
**Age groups**			
<60 years old	42	Deficient (<30 ng/mL)	19.39 ± 6.26
	57	Sufficient (≥30 ng/mL)	49.41 [24.70]
≥60 years old	5	Deficient (<30 ng/mL)	24.34 ± 3.75
	15	Sufficient (≥30 ng/mL)	49.00 ± 12.94
**Cancer types**			
Hematologic	3	Deficient (<30 ng/mL)	23.06 ± 2.39
	6	Sufficient (≥30 ng/mL)	50.87 [32.86]
Solid	44	Deficient (<30 ng/mL)	19.69 ± 6.33
	66	Sufficient (≥30 ng/mL)	49.22 [22.79]
**Chemotherapy**			
Yes	37	Deficient (<30 ng/mL)	20.43 ± 6.29
	59	Sufficient (≥30 ng/mL)	49.41 [21.65]
No	10	Deficient (<30 ng/mL)	17.97 ± 5.72
	13	Sufficient (≥30 ng/mL)	46.42 [32.44]
**Chemotherapy regimen**			
Single	11	Deficient (<30 ng/mL)	17.96 ± 6.52
	17	Sufficient (≥30 ng/mL)	49.60 ± 14.22
Combination	18	Deficient (<30 ng/mL)	21.28 ± 6.69
	27	Sufficient (≥30 ng/mL)	51.59 [29.42]
**Time since last chemotherapy**			
≤6 months	8	Deficient (<30 ng/mL)	25.32 ± 3.25
	21	Sufficient (≥30 ng/mL)	53.60 ± 12.47
>6 months	29	Deficient (<30 ng/mL)	19.08 ± 6.28
	38	Sufficient (≥30 ng/mL)	46.23 [22.06]
**History of comorbidities**			
Yes	19	Deficient (<30 ng/mL)	19.73 ± 7.18
	18	Sufficient (≥30 ng/mL)	52.75 [32.57]
No	28	Deficient (<30 ng/mL)	20.03 ± 5.56
	54	Sufficient (≥30 ng/mL)	47.74 [22.24]

25(OH)D, 25-hydroxy-vitamin D; IQR, interquartile range; SD, standard deviation; COVID-19, coronavirus disease 2019.

In Table 3, the S-RBD IgG level was significantly higher in the sufficient group (median = 414.07 [1,441.83] AU/mL) compared to the deficient group (median = 91.56 [652.00] AU/mL) among subjects consuming vitamin D_3_ daily supplementation (*p*-value = 0.049).

**TABLE 3:** The comparison between serum 25(OH)D levels and anti-SARS-CoV-2 antibody (anti-SARS-CoV-2 S-RBD IgG and NAb) levels in subjects subgroup by vitamin D_3_ supplementation daily consumption.

Vitamin D_3_ daily supplementation	*N*	Serum 25(OH)D level (ng/mL)		S-RBD IgG level (AU/mL)		NAb level (AU/mL)	
		Categories	Averages, in median [IQR] or mean ± SD	Median [IQR]	Significance	Median [IQR]	Significance
Yes	22	Deficient (<30 ng/mL)	21.00 ± 5.36	91.56 [652.00]	**0.049^b^**	33.51 [208.67]	0.066^b^
	61	Sufficient (≥30 ng/mL)	52.66 ± 25.99	414.07 [1441.83]		149.71 [410.68]	
No	25	Deficient (<30 ng/mL)	18.94 ± 6.81	227.35 [581.67]	0.357^a^	129.03 [250.19]	0.303^a^
	11	Sufficient (≥30 ng/mL)	38.04 ± 6.37	102.65 [427.77]		35.42 [156.24]	

^a^Analyzed using *t*-test after transformation.

^b^Analyzed using Mann-Whitney *U* test.

25(OH)D, 25-hydroxy-vitamin D; S-RBD IgG, antibodies against receptor binding domain of SARS-CoV-2 spike protein; NAb, neutralizing antibody; IQR, interquartile range; SD, standard deviation.

Bold value denotes statistical significance.

In Table 4, among subjects treated without chemotherapy, the anti-SARS-CoV-2 S-RBD IgG had a significantly moderate positive correlation with the 25(OH)D level (*p*-value = 0.03; *R* = 0.588). The NAb levels also demonstrated a significantly moderate positive correlation with 25(OH)D level (*p*-value = 0.005; *R* = 0.561). These significantly positive correlation indicated that S-RBD IgG and NAb levels are directly related to the levels of 25(OH)D.

**TABLE 4:** The correlation between serum 25(OH)D levels and anti-SARS-CoV-2 antibody (anti-SARS-CoV-2 S-RBD IgG and NAb) among subjects received chemotherapy and non-chemotherapy.

Chemotherapy	Anti-SARS-CoV-2 antibody level	*N*	Median [IQR] (AU/mL)	Serum 25(OH)D level			
				Median [IQR] or mean ± SD (ng/mL)	Significance	Correlation coefficient	Interpretation
Yes	S-RBD IgG^a^	96	275.53 [600.26]	38.94 ± 18.15	0.811	0.025	No significant correlation
	NAb^b^	96	126.74 [224.88]		0.839	0.014	No significant correlation
No	S-RBD IgG^a^	23	244.94 [783.20]	33.80 [44.50]	0.030	0.588	**Moderate correlation**
	NAb^a^	23	129.03 [256.58]		0.005	0.561	**Moderate correlation**

^a^Analyzed using Pearson’s correlation test after transformation into logX (AU/mL).

^b^Analyzed using Kendall’s correlation test.

25(OH)D, 25-hydroxy-vitamin D; S-RBD IgG, antibodies against receptor binding domain of SARS-CoV-2 spike protein; NAb, neutralizing antibody; IQR, interquartile range; SD, standard deviation.

Bold values denotes statistical significance.

**FIGURE 1:**
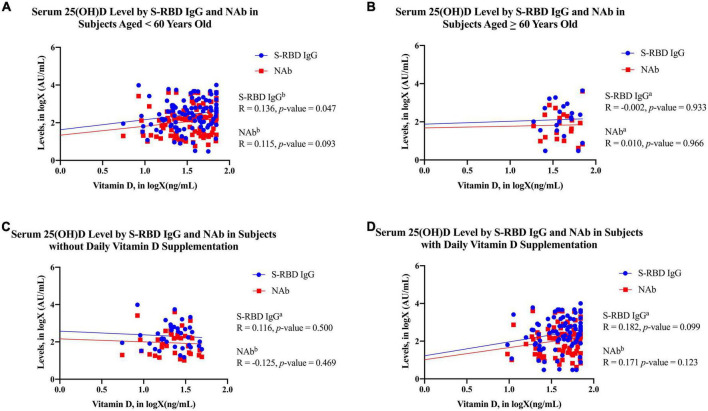
The scatter plots of 25(OH)D in logX (ng/mL) by S-RBD IgG in logX (AU/mL) and NAb in logX (AU/mL) in each subject characteristics. **(A)** Subjects less than 60 years old; **(B)** subjects over 60 years old; **(C)** subjects without daily vitamin D_3_ supplementation; **(D)** subjects with daily vitamin D_3_ supplementation. The blue dots and line in the graph present the S-RBD IgG in logX (AU/mL), whereas the red squares and line present the NAb in logX (AU/mL). ^a^Analyzed using Pearson’s correlation test after transformation into logX (AU/mL). ^b^Analyzed using Kendall’s correlation test. 25(OH)D, 25-hydroxy-vitamin D; S-RBD IgG, antibodies against receptor binding domain of SARS-CoV-2 spike protein; NAb, neutralizing antibody.

In Figure 1A, the 25(OH)D level was positively correlated with the S-RBD IgG levels among subjects younger than 60 years old (*p*-value = 0.047; *R* = 0.136). However, the serum (Figures 1A–D) 25 (OH)D level was not correlated with the S-RBD IgG level among subjects older than 60 years old (*p*-value = 0.933; *R* = 0.136).

Separate hematologic malignancies analysis could not be conducted due to the unfulfilled minimum sample size for some subgroups for Pearson’s or Kendall’s correlation test (Tables 5, 6). Therefore, the subjects were still analyzed as a whole in the study to represent the general cancer population with both hematologic and solid malignancies.

**TABLE 5:** The comparison between serum 25(OH)D levels and anti-SARS-CoV-2 antibody (anti-SARS-CoV-2 S-RBD IgG and NAb) levels in subjects subgroup by hematologic malignancies.

Vitamin D_3_ daily supplementation	*N*	Serum 25(OH)D level (ng/mL)		S-RBD IgG level (AU/mL)		NAb (AU/mL)	
		Categories	Averages, in median [IQR] or mean ± SD	Averages, in median [IQR] or mean ± SD	*P*-value	Averages, in median [IQR] or mean ± SD	*P*-value
Yes	2	Deficient (<30 ng/mL)	–	–	–	–	–
	6	Sufficient (≥30 ng/mL)	50.87 [32.86]	2135.81 ± 2256.66		1204.68 ± 1578.43	
No	1	Deficient (<30 ng/mL)	–	–	–	–	–
	0	Sufficient (≥30 ng/mL)	–	–		–	

25(OH)D, 25-hydroxy-vitamin D; S-RBD IgG, antibodies against receptor binding domain of SARS-CoV-2 spike protein; NAb, neutralizing antibody; IQR, interquartile range; SD, standard deviation.

**TABLE 6:** The correlation between serum 25(OH)D levels and anti-SARS-CoV-2 antibody (anti-SARS-CoV-2 S-RBD IgG and NAb) among hematologic malignancies subjects received chemotherapy and non-chemotherapy.

Chemotherapy	Anti-SARS-CoV-2 antibody level	*N*	Median [IQR] or mean ± SD (AU/mL)	Serum 25(OH)D level		
				Median [IQR] or mean ± SD (ng/mL)	Significance	Correlation coefficient
Yes	S-RBD IgG^a^	7	15.62 [293.86]	34.92 ± 14.27	0.598	−0.244
	NAb^a^	7	9.32 [659.13]		0.452	−0.342
No	S-RBD IgG	2	–	–	–	–
	NAb	2	–		–	–

^a^Analyzed using Pearson’s correlation test after transformation into logX (AU/mL).

25(OH)D, 25-hydroxy-vitamin D; S-RBD IgG, antibodies against receptor binding domain of SARS-CoV-2 spike protein; NAb, neutralizing antibody; IQR, interquartile range; SD, standard deviation.

Some multivariate regression models were then formulated to calculate the level of S-RBD IgG and NAb based on the available variables:


$$S-R\,B\,D\,I\,g\,G=b\,0+b\,1^{\prime }\,A\,g\,e+b\,2^{\prime }\,C\,a\,n\,c\,e\,r\,C\,l\,a\,s\,s\,i\,f\,i\,c\,a\,t\,i\,o\,n$$

$$\;\;+b\,3^{\prime }\,T\,i\,m\,e\,s\,i\,n\,c\,e\,l\,a\,s\,t\,c\,h\,e\,m\,o\,t\,h\,e\,r\,a\,p\,y$$

$$\;\;+b\,4^{\prime }\,H\,i\,s\,t\,o\,r\,y\,o\,f\,c\,o\,m\,o\,r\,b\,i\,d\,i\,t\,i\,e\,s$$

$$\;\;+b\,5^{\prime }\,H\,i\,s\,t\,o\,r\,y\,o\,f\,C\,O\,V\,I\,D-19\,i\,n\,f\,e\,c\,t\,i\,o\,n$$

$$\;\;+b\,6^{\prime }\,V\,i\,t\,a\,m\,i\,n\,D\,s\,u\,p\,p\,l\,e\,m\,e\,n\,t\,a\,t\,i\,o\,n$$

$$\;\;+b\,7^{\prime }\,S\,e\,r\,u\,m\,v\,i\,t\,a\,m\,i\,n\,D\,l\,e\,v\,e\,l$$

$$N\,A\,b=b\,0+b\,1^{\prime }\,A\,g\,e+b\,2^{\prime }\,C\,a\,n\,c\,e\,r\,C\,l\,a\,s\,s\,i\,f\,i\,c\,a\,t\,i\,o\,n$$

$$\;\;+b\,3^{\prime }\,T\,i\,m\,e\,s\,i\,n\,c\,e\,l\,a\,s\,t\,c\,h\,e\,m\,o\,t\,h\,e\,r\,a\,p\,y$$

$$\;\;+b\,4^{\prime }\,H\,i\,s\,t\,o\,r\,y\,o\,f\,c\,o\,m\,o\,r\,b\,i\,d\,i\,t\,i\,e\,s$$

$$\;\;+b\,5^{\prime }\,H\,i\,s\,t\,o\,r\,y\,o\,f\,C\,O\,V\,I\,D-19\,i\,n\,f\,e\,c\,t\,i\,o\,n$$

$$\;\;+b\,6^{\prime }\,V\,i\,t\,a\,m\,i\,n\,D\,s\,u\,p\,p\,l\,e\,m\,e\,n\,t\,a\,t\,i\,o\,n$$

$$\;\;+b\,7^{\prime }\,S\,e\,r\,u\,m\,v\,i\,t\,a\,m\,i\,n\,D\,l\,e\,v\,e\,l$$

The multivariate regression analysis demonstrated that there was no significant correlation between anti-SARS-CoV-2 S-RBD IgG and NAb with age, cancer classification, time since last chemotherapy, history of comorbidities, daily vitamin D3 supplementation, and serum 25(OH)D levels (Supplementary Tables 1, 2). However, these results might not represent the actual correlation due to our data not meeting the criteria to conduct the multivariate regression analysis. Despite the fact that the serum 25(OH)D level is a ratio numerical variable, multivariate regression analysis was unable to be performed due to the data being non-normally distributed despite transformation. Furthermore, the variances were not equal among the variables.

## Discussion

Recent research and development have led to the creation of inactivated vaccines, viral vector, and mRNA vaccines for SARS-CoV-2 in an effort to combat the virus (12). However, the correlation between serum 25(OH)D and the efficacy of COVID-19 vaccines as measured by S-RBD IgG and NAb among cancer patients remain unclear.

Based on previous studies, Indonesia has a high prevalence of vitamin D deficiency (60%) and low 25(OH)D levels (mean = 21 ng/mL) (37–42). Since the amounts of 25(OH)D can be affected by skin pigmentation, vitamin D deficiency was widespread in Indonesia (43). According to the Fitzpatrick skin phototype classification, based on the skin’s sensitivity to UV radiation, Asians (including Indonesians) have Fitzpatrick phototype IV (medium to dark brown skin) or phototype V (dark brown skin) (43, 44). The presence of melanin in the skin inhibits the conversion of 7-dehydrocholesterol to previtamin D_3_, rendering the vitamin D production extremely dependent on the melanin concentration. This happens because melanin absorbs and scatters UVR-B. Recent research has shown that greater melanin levels in darker skin may reduce vitamin D absorption. Therefore, vitamin D production is suppressed in people with dark skin compared to people with lighter skin (43, 45–47).

Furthermore, a recent study reported that 72% of cancer patients were suffering from vitamin D deficiency (48–52). In a meta-analysis of prospective cohort studies, it was reported that subjects with better vitamin D status reduced the cancer mortality risk by 19%. Cancer mortality was also reduced 2% for every 8 ng/mL (20 nmol/L) of 25(OH)D concentration increment (48, 53). Common risk factors for vitamin D deficiency in the cancer population were palliative care management and adjuvant chemotherapy administration (48, 52). Studies revealed that vitamin D has antineoplastic effect. Vitamin D receptors are expressed extensively throughout the body, which further encourages the antineoplastic behavior of vitamin D (48, 54). Apoptosis, immunomodulatory effects, and antiproliferative effects are produced as a result of transcriptional activity and target gene suppression following the binding of vitamin D to the vitamin D receptors. These actions have the potential to contribute to a decrease in the catastrophic cancer and metastatic disease incidence rates (29, 48, 54, 55).

Interestingly, in this study, the median level of 25(OH)D serum was 36.36 [30.30] ng/mL, which indicated that the cancer patients had reached a sufficient vitamin D status according to the Endocrine Society (Table 2) (36). Based on the current evidence, sunlight exposure and vitamin D_3_ supplementation are the two key factors in determining 25(OH)D levels (56, 57). Indonesia consists of more than 17,000 islands that are split by the equator, resulting in a tropical climate with an adequate sunlight and temperatures that are generally stable throughout the year (42). Ultraviolet B (UVB) light with a wavelength between 290 and 315 nm induces 7-dehydrocholesterol in the skin to be converted into previtamin D. Heat isomerization transforms this previtamin D into the active form of vitamin D. The 25(OH)D is an efficient marker of vitamin D status, which is produced when the liver metabolizes the vitamin D from the skin (36, 56). Furthermore, the subjects in this study were already educated with the benefits of daily vitamin D_3_ supplementation. A meta-analysis performed by Tripkovic et al. revealed that vitamin D_3_ (cholecalciferol) was more effective in boosting serum 25(OH)D levels than vitamin D_2_ (ergocalciferol), hence the reason that vitamin D_3_ was chosen for daily supplementation in this study (58). Subjects who participated in this study also made tremendous efforts to improve their immune systems by modifying their vitamin D status. As a result, sufficient vitamin D status among cancer patients in this study was primarily accomplished through sun exposure and daily vitamin D_3_ supplementation.

The level of anti-SARS-CoV-2 S-RBD IgG was significantly higher in the group that had daily consumption of vitamin D_3_ and sufficient vitamin D status as compared to the group that had deficient vitamin D status (Table 3). Our finding was supported by Chel et al., who conducted a randomized clinical trial study that evaluated the effect of vitamin D_3_ oral doses of 600 IU/day, 4,200 IU/week, and 18,000 IU/month on the vitamin D status. The daily vitamin D_3_ was more efficacious than weekly and monthly administrations (59). These finding was consistent with the Endocrine Society that the vitamin D could only perform its function when serum 25(OH)D levels reached 30 ng/mL (75 nmol/L) or higher (36, 38). Vitamin D has major effects on both the innate and adaptive immune systems through its biological activities (12, 27). To convert 25(OH)D to 1,25(OH)2D, circulating levels of 25(OH)D must be at least 30 ng/mL (75 nmol/L) (27, 31, 60, 61). After 1,25(OH)2D has been produced, it modulates both the innate and adaptive immune systems through autocrine and paracrine mechanisms. Several studies suggest that vitamin D may affect immune activity through a non-genomic mechanism by maintaining endothelium membranes (27, 62). The majority of evidence to date emphasizes the importance of adequate 25(OH)D levels in modifying immunological function (12, 27).

Vitamin D_3_ supplementation may improve COVID-19 vaccination efficacy by boosting immunological responses. The vaccine is identified as an antigen to both CD8+ and CD4+ T cells via an antigen-presenting cell (APC). After being activated by THαβ cytokines, the CD8+ T lymphocytes are stimulated and have the capability to eliminate infected cells. An adequate vitamin D_3_ supplementation may be advantageous in optimizing this mechanism. THαβ cytokines promote the B cells differentiation. After being activated, the B cells have the capability to produce NAb. Antibody production can be enhanced by vitamin D in a T-cell-dependent B-cell mechanism (12, 63, 64). As a result, maintaining serum 25(OH)D levels in the blood above 30 ng/mL with daily vitamin D_3_ supplementation can improve the efficacy of COVID-19 vaccines.

To the best of our knowledge, this is the first study that found a significant positive correlation between 25(OH)D levels and anti-SARS-CoV-2 S-RBD IgG and NAb levels in cancer patients who did not receive chemotherapy (Table 4). In contrast, Jolliffe et al. found that 25(OH)D had no effect on the protective efficacy or immunogenicity of the SARS-CoV-2 vaccination when given to adults who had deficient vitamin D status [mean 25(OH)D levels of 15.9 ng/mL (39.9 nmol/L)]. All participants had 25(OH)D concentrations below 30 ng/mL (75 nmol/L) (65). Another study by Chillon et al. found that 25(OH)D levels did not correlate with anti-SARS-CoV-2 IgG levels in healthy subjects with deficient vitamin D status [median 25(OH)D levels less than 30 ng/mL (75 nmol/L)] during four sequential measurements (66). However, vitamin D could only perform its function when blood 25(OH)D serum levels exceeded 30 ng/mL (75 nmol/L) (36, 38). In our study, the median 25(OH)D level was 36.36 ng/mL (90 nmol/L), indicating that the subjects had sufficient vitamin D status (Table 2). A systematic review by Corti et al. reported that patients with cancer had a lower seroconversion rate than healthy subjects after COVID-19 vaccine vaccination (25). This was in contrast with our study, in which the cancer patients successfully reached high seropositivity of anti-SARS-CoV-2 S-RBD IgG and NAb. A sufficient vitamin D status led to a significantly positive correlation between vitamin D and anti-SARS-CoV-2 S-RBD IgG and NAb antibodies. Immune cells, including dendritic cells, macrophages, monocytes, and lymphocytes, express the VDR and metabolizing enzymes (27, 30, 31). Vitamin D has been proven in studies to have major biologic effects on the innate and adaptive immune systems (12, 27). 1,25-dihydroxyvitamin D inhibits NF-κB p65 activation and directly controls the inflammatory cytokines that rely on NF-κB activity in macrophages (12, 27, 67). In the circulation, dendritic cells, B cells, and T cells utilize 25D via intracrine conversion to bioactive 1,25D by expressing the VDR and the enzyme CYP27B1 (1α-hydroxylase) (12, 27, 68).

On the other hand, the COVID-19 vaccination elicits a THαβ response that is protective against the virus (12, 69). THαβ produces IgG1 B cells, CD4 T cells, CD8 T cells, and NK cells. In B cells, the isotype transition from IgM to IgG can be facilitated by follicular helper T cells (ThFH). Antibody-dependent cellular cytotoxicity (ADCC) and apoptosis of the infected cells can be mediated by CD8 T cells and NK cells through the perforins and granzymes. Long-term memory B cells, CD4 T cells, and CD8 T cells are activated and established due to vaccines (12, 70–72). These intriguing findings provide the explanation for the hitherto unexplained correlation between vitamin D and the efficacy of vaccination against SARS-CoV-2.

In subjects who did not receive chemotherapy, serum 25(OH)D levels demonstrated significantly positive correlations with both anti-SARS-CoV-2 S-RBD IgG and NAB. Several studies found that patients receiving chemotherapeutic regimens such as anthracycline and taxane had significantly lower 25(OH)D levels (73, 74). Cytostatic such as paclitaxel and cyclophosphamide, which are ligands of the pregnane X receptor and induce 24-hydroxylase, can speed up the enzymatic degradation of 25(OH)D and 1,25(OH)2D during chemotherapy (73, 75, 76). Chemotherapy may impair vitamin absorption due to dysgeusia and subclinical mucositis (73, 77, 78). Docetaxel has been associated with a variety of unpleasant adverse events, including those that manifest on the skin and in the sense of taste (73, 77). On the other hand, vitamin D is known as the “sunshine vitamin” because it is synthesized in the skin as a response to sunlight (36). However, the cutaneous adverse events induced by chemotherapy may hinder vitamin D synthesis. Chemotherapy may eventually activate CYP3A4 and other metabolizing enzymes, resulting in the conversion of 25(OH)D into inactive molecules like 24,25OH vitamin (77). Additionally, we also found that S-RBD IgG positively correlated with 25(OH)D levels among subjects younger than 60 years old (Figure 1A). The production of 1,25(OH)2D, an active form of vitamin D, is altered by aging (79, 80). Serum 1,25(OH)2D levels are maintained partially by secondary hyperparathyroidism despite a 50% reduction in production due to age-related decrease in renal function (79, 81). The development of 1,25(OH)2D is inhibited by vitamin D deficiency due to its reliance on a sufficient supply of the vitamin D substrate for vitamin D production (79, 82). Aging also decreases vitamin D synthesis in the skin through reducing 7-dehydrocholesterol concentration in the epidermis and UV light sensitivity, resulting in a 50% reduction in previtamin D_3_ (79, 83, 84). However, a pilot study by Borecka et al. found that older subjects with increased vitamin D consumption had higher serum 25(OH)D levels than younger subjects did (85). Thereby, we propose daily vitamin D_3_ supplementation in older subjects can enhance the serum 25(OH)D levels, which boosts the COVID-19 vaccine efficacy.

To combat SARS-CoV-2, vaccination has been and will continue to be a vital component. However, recent evidence indicates that vaccination alone would not be sufficient to stop the spread of SARS-CoV-2. The situation may worsen dramatically if new viral variants emerge that are resistant to all or even most existing vaccinations (39, 86, 87). As a result, our findings strongly suggest that daily vitamin D3 supplementation and maintaining serum 25(OH)D levels above 30 ng/mL for all cancer patients will increase the efficacy of the vaccination. The ultimate goal here is to save more lives. Considering the social and political implications, it will lessen the requirement for additional contact restrictions and lockdowns. Vitamin D is not expensive and, in combination with vaccinations, offers a promising chance to limit the spread of SARS-CoV-2, which might save trillions of dollars worldwide (39).

Our findings with regards to vitamin D enhancing the efficacy of COVID-19 vaccines are consistent with this evidence. The heterogeneous cancer types may be a limiting factor here, potentially missing a substantial finding. However, we can conclusively state that daily supplementation of vitamin D_3_ enhances the efficacy of COVID-19 vaccines. These interesting findings indicate the need for larger studies, powered to enable a more accurate representation of the cancer population, to further evaluate our understanding of vitamin D in enhancing the efficacy of COVID-19 vaccines.

Furthermore, the authors suggest the need for further study investigating the optimal dose for daily supplementation of vitamin D_3_ in order to enhance the COVID-19 vaccine’s efficacy among cancer patients. The recommended daily dose of vitamin D_3_ among cancer patients has not been established (88). The Endocrine Society’s Clinical Practice Guideline recommends 1,500–2,000 IU per day for all healthy adults to keep 25(OH)D levels above the optimal level of 30 ng/mL (38, 88–90). The 2011 US Institute of Medicine suggested a dose of 600 IU per day for adults aged up to 70 years and 800 IU per day for older adults. The tolerable upper limit is 4,000 IU per day. Beyond this dose, the risk of toxic effects is increased (38, 91). Hence, further studies to determine an optimal daily dose for vitamin D_3_ are crucial to enhance the efficacy COVID-19 vaccine and prevent toxicity from excessive doses.

The strength of the study was the fact that it was conducted among subjects with various types of cancer. Thus, it was expected to be able to represent the general cancer population. However, there are limitations that should be considered in the interpretation of our results. Due to the Indonesian government’s policy requiring rapid third-dose vaccination for all Indonesian citizens, we did not observe the pre- and post-vaccine serum 25(OH)D level and its association with the COVID-19 antibody. Therefore, it became unreachable to recruit further subjects that had naïve antibody. Second, this is a cross sectional study. Thus, our finding should be confirmed by further interventional trials such as randomized clinical trial.

## Conclusion

We found that the median levels of serum 25(OH)D among cancer subjects reached sufficient vitamin D status. The anti-SARS-CoV-2 S-RBD IgG and NAb also reached high seropositivity. The S-RBD IgG levels were significantly higher in the sufficient vitamin D group compared to the deficient vitamin D group among subjects who consistently consume vitamin D_3_ daily supplementation. The serum 25(OH)D levels demonstrated a significantly positive correlation with both anti-SARS-CoV-2 S-RBD IgG and NAb levels among non-chemotherapy subjects. The S-RBD IgG levels were found to be positively correlated with serum 25(OH)D levels among subjects younger than 60 years old. Therefore, we strongly suggest cancer patients to combine COVID-19 vaccinations with daily vitamin D_3_ supplementation to ensure blood levels above 30 ng/mL (75 nmol/L), as this will significantly improve their immune system.

## Data availability statement

The original contributions presented in this study are included in the article/Supplementary material, further inquiries can be directed to the corresponding author.

## Ethics statement

Ethical approval for this study was granted by the Ethics Committee of the Faculty of Medicine, Universitas Indonesia (ethical approval number: KET 999/UN2.F1/ETIK/PPM.00.02/2021). This research was performed in accordance with the Declaration of Helsinki. The patients/participants provided their written informed consent to participate in this study.

## Author contributions

AR and AI: conceptualization and methodology. DP and BR: data curation and funding acquisition. AR, RB, and SJ: formal analysis. AR, AI, and DP: investigation and resources. RB and SJ: project administration. BR, RB, and SJ: software and writing—original draft. AR, BR, RB, and SJ: writing—reviewing and editing. All authors contributed to the article and approved the submitted version.
