# Supplementary material for: The correlation between serum 25-hydroxy-vitamin D levels and anti-SARS-CoV-2 S-RBD IgG and neutralizing antibody levels among cancer patients receiving COVID-19 vaccines
Source: Front Nutr. 2022 Dec 13;9:1066411. doi: 10.3389/fnut.2022.1066411 (PMC9792493; doi:10.3389/fnut.2022.1066411)
Supplement: Supplementary file 1 [file Table_1.DOCX]

Supplementary Material

The correlation between serum 25-hydroxy-vitamin D levels and anti-SARS-CoV-2 S-RBD IgG and neutralizing antibody levels among cancer patients receiving COVID-19 vaccines

Andhika Rachman^1*^, Anggraini Iriani^2^, Dimas Priantono^1^, Bayu Bijaksana Rumondor^3^, Rachelle Betsy^3^, Samuel Juanputra^3^

^1^Division of Hematology and Medical Oncology, Department of Internal Medicine, Dr. Cipto Mangunkusumo General Hospital - Faculty of Medicine Universitas Indonesia, Jakarta, Indonesia

^2^Department of Clinical Pathology, Yarsi University, Jakarta, Indonesia

^3^Department of Internal Medicine, Dr. Cipto Mangunkusumo General Hospital - Faculty of Medicine Universitas Indonesia, Jakarta Indonesia.

*** Correspondence:**Andhika Rachman, MD, PhD.

Division of Hematology and Medical Oncology, Department of Internal Medicine, Dr. Cipto Mangunkusumo General Hospital – Faculty of Medicine, Universitas Indonesia. Jl. Pangeran Diponegoro No.71, RW.5, Kec. Senen, Central Jakarta, Jakarta 10430, Indonesia.

Email: [andhikarachman@gmail.com](mailto:andhikarachman@gmail.com) | Phone: +62813-9862-0570Supplementary Data

**Supplementary Table 1.** The multivariate regression analysis of S-RBD IgG

| **Variable** | **Coefficient** $\boldsymbol{(}\boldsymbol{\beta)}$ | ***P*** |
| --- | --- | --- |
| Constant | 2208.735 | .055 |
| History of COVID-19 infection | 672.446 | .146 |
| Cancer classification | 672.446 | .660 |
| Time since last chemotherapy | -237.700 | .184 |
| History of comorbidity | 54.122 | .891 |
| Daily vitamin D_3_ supplementation | -248.819 | .578 |
| Serum 25(OH)D level | -16.559 | .154 |

Abbreviation: 25(OH)D, 25-hydroxy-vitamin D; S-RBD IgG, antibodies against receptor binding domain of SARS-CoV-2 spike protein

**Supplementary Table 2.** The multivariate regression analysis of NAb

| **Variable** | **Coefficient** $\boldsymbol{(\beta)}$ | ***P*** |
| --- | --- | --- |
| Constant | 1231.767 | .039 |
| History of COVID-19 infection | 486.139 | .045 |
| Cancer classification | -122.024 | .749 |
| Time since last chemotherapy | -101.895 | .272 |
| History of comorbidity | -90.946 | .658 |
| Daily vitamin D_3_ supplementation | -238.167 | .306 |
| Serum 25(OH)D level | -9.229 | .127 |

Abbreviation: 25(OH)D, 25-hydroxy-vitamin D; NAb, neutralizing antibody
